# Identification of the tetraspanin gene family in sugarcane and its response to sugarcane mosaic virus infection

**DOI:** 10.3389/fpls.2025.1684431

**Published:** 2025-10-27

**Authors:** Zhiyuan Cui, Yifei Li, Kang Zeng, Zongtao Yang, Quanxin Yu, Haoming Liu, Zhenhe Zhan, Hai Zhang, Guoqiang Huang, Jingsheng Xu

**Affiliations:** National Engineering Research Center for Sugarcane, Key Laboratory of Sugarcane Biology and Genetic Breeding, Ministry of Agriculture and Rural Affairs, College of Agriculture, Fujian Agriculture and Forestry University, Fuzhou, Fujian, China

**Keywords:** sugarcane, sugarcane mosaic virus, tetraspanin, potyvirus, 6K2 protein

## Abstract

**Introduction:**

Sugarcane mosaic virus (SCMV, *Potyvirus*) causes mosaic diseases and seriously threatens sugarcane production. Potyviral 6K2 protein plays a key role in viral infections. We previously screened a tetraspanin (TET)-like protein that interacts with SCMV-6K2 from a sugarcane cDNA yeast library. Although TETs have been extensively studied in response to viral infections in animals, the TET gene family in sugarcane and its role in SCMV infections remain largely unknown. This study aimed to identify the *TET* genes in sugarcane and determine their response to SCMV infection.

**Methods:**

We employed genome-wide identification, phylogenetic analysis, real-time quantitative PCR (RT-qPCR), subcellular localization, and multiple protein–protein interaction assays to characterize TETs and their interactions with viral 6K2 proteins.

**Results:**

We identified 35, 113, 73, and 17 TETs in the genomes of *Saccharum* sp*ontaneum*, sugarcane cultivar R570, sugarcane cultivar Xintaitang 22 (XTT22), and *Nicotiana benthamiana*, respectively. Phylogenetic tree analysis classified the TETs into nine distinct groups. Nine TET genes were cloned from XTT22 and designated *ScTET2*, *ScTET8*, *ScTET13*, *ScTET23*, *ScTET34*, *ScTET55*, *ScTET67*, *ScTET78*, and *ScTET96*. RT-qPCR demonstrated the differential expression of these genes following SCMV infection. Furthermore, subcellular localization assays revealed that they were mainly localized to the plasma membrane (PM), except for ScTET2 and ScTET8, which were localized in the cytoplasm and formed irregular spherical structures of different sizes. Yeast two-hybrid (Y2H), bimolecular fluorescent complementation, and luciferase complementation assays revealed extensive interactions between the ScTETs and SCMV-6K2, primarily in the PM. Y2H assays also showed that TETs of *Arabidopsis* and *N. benthamiana* extensively interacted with the 6K2 protein of turnip mosaic virus.

**Discussion:**

This study reveals a potential mechanism by which potyviruses employ 6K2 to interact with TETs to establish infection in host plants, thus highlighting potential molecular targets for engineering sugarcane resistance against SCMV.

## 1 Introduction

Potyviruses represent the largest group of plant viruses and cause heavy yield losses in many crops worldwide (Urcuqui-Inchima et al., 2001; Revers and García, 2015; Yang et al., 2024). Potyviruses are flexuous rod-shaped particles at 680–900 nm long and 11–20 nm wide, and they contain approximately 10 kb of single-stranded positive-sense RNA that encodes two polyproteins (Urcuqui-Inchima et al., 2001; Yang et al., 2021; Pollari et al., 2024). These two polyproteins hydrolyze into 11 mature proteins: P1, HC-Pro, P3, P3N-PIPO, 6K1, CI, 6K2, Vpg, NIa-Pro, NIb, and CP (Valli et al., 2007; Chung et al., 2008; Cheng et al., 2017, 2020; Xiao et al., 2022; Hýsková et al., 2024). Among these, the 6K2 single-transmembrane protein localized in the endoplasmic reticulum (ER) participates in multiple biological processes during potyvirus infection (Xue et al., 2023; Zhang et al., 2024a; 2024). The 6K2 protein can induce the rearrangement of ER at ER exit sites (ERESs) to form virus replication complexes (Grangeon et al., 2013; Jiang et al., 2015; Cabanillas et al., 2018; Zhang et al., 2019; Xie et al., 2021; Solovyev et al., 2022) and fuse with other endomembrane systems, such as the outer membrane of chloroplasts, to facilitate efficient replication. Notably, it plays a role in the intra- or intercellular movement as well as long-distance transport of the virus (Wan et al., 2015; Movahed et al., 2019; Chai et al., 2020; He et al., 2023b) and immune responses, including reactive oxygen species (ROS) burst and autophagy (Wang, 2015; Hafrén et al., 2018; Li et al., 2020; Lin et al., 2024; Zhang et al., 2024; He et al., 2025; Rui et al., 2025). Interestingly, overexpressing of turnip mosaic virus (TuMV) 6K2 in *Arabidopsis* and *Nicotiana benthamiana* promotes salicylic acid accumulation and resistance to drought stress (Prakash et al., 2023). Given the significant role of 6K2 in potyvirus infection, we previously screened a sugarcane cDNA yeast library using the 6K2 protein of SCMV as bait and identified the tetraspanin (TET)-like protein ScTSPAN18 (Zhang H. et al., 2019).

TETs are evolutionarily conserved integral membrane proteins in cellular organisms (Hemler, 2003; Wang et al., 2012a; Green et al., 2019; Zhang et al., 2025a). TETs are composed of four transmembrane domains, one small extracellular loop, and one highly variable large extracellular loop (LEL) (Boucheix and Rubinstein, 2001; Hemler, 2003; Kovalenko et al., 2005; Reimann et al., 2017). In animals, LEL contains a conserved CCG motif, whereas in plants, it contains a GCCK/RP motif (Seigneuret et al., 2001; Huang et al., 2005; Wang et al., 2012a; Boavida et al., 2013). TETs can interact with themselves, other TETs, or other ligand proteins on the plasma membrane (PM) to form TET-enriched microdomains (TEMs), which are involved in signaling, cell adhesion, migration, proliferation, differentiation, fundamental immune response, and PM repair (Le Naour et al., 2000; Miyado et al., 2000; Boucheix and Rubinstein, 2001; Hemler, 2001; Charrin et al., 2009; Umeda et al., 2020; Huang et al., 2022; Zhang et al., 2025b). In mammals, TETs such as CD9, CD81, CD63, CD82, and CD151 are extensively involved in viral infections (Fast et al., 2017; Florin and Lang, 2018; Zhang et al., 2025b). CD9, CD63, and CD81 are components of extracellular vesicles (EVs) and respond to infection with the human immunodeficiency virus, Lujo virus, hepatitis B virus, or herpes simplex virus 1 (Florin and Lang, 2018; Ghossoub et al., 2020; Mathieu et al., 2021; Ninomiya et al., 2021; Poveda et al., 2022; Zhang et al., 2025b).

Plant TETs are involved in plant development and growth, including cell fate determination, hormonal regulation, plasmodesmata gating, and signaling (Cnops et al., 2006; Wang et al., 2015; Reimann et al., 2017). For instance, *TET* mutants have abnormal leaves or roots (Cnops et al., 2006; Qin et al., 2024; Zimmerman et al., 2024). Plant TETs are also involved in response to biotic and abiotic stresses and mutualistic interactions (Parra-Aguilar et al., 2023; Chen et al., 2025). In rice (*Oryza sativa*), OsTET5 regulates drought resistance by controlling ROS burst and ionic homeostasis (Mani et al., 2025), whereas in potato (*Solanum tuberosum*), StTET8 act as a positive immune regulator that inhibits *Phytophthora infestans* infection (Guo et al., 2022). Interestingly, PsTET1 and PsTET3 of soybean (*Glycine max*) root rot pathogen *Phytophthora sojae* are recognized by *N. benthamiana*, where they elicit immune responses (Zhu et al., 2023). In *Capsicum*, expression of the *TET8-like* gene is strongly correlated with the accumulation of capsicum chlorosis virus (CaCV) (Cnops et al., 2006; Wang et al., 2015; Reimann et al., 2017). In *Arabidopsis*, AtTET3 plays a key role in the cell-to-cell movement of cucumber mosaic virus (CMV) (Zhu et al., 2022). Plant TETs are also involved in the formation and signaling of EVs (Cui et al., 2020; He et al., 2021; Chen et al., 2022; Ruf et al., 2022; He et al., 2023a; Gao et al., 2024; Chen et al., 2025). *AtTET8* and *AtTET9* in *Arabidopsis* mediate the transport of EVs carrying RNA, including host-derived small RNAs (sRNAs), to fungal cells, thereby reducing fungal infection (Cai et al., 2018). Conversely, pathogenic fungi can also use EVs to deliver sRNAs into plant cells (He et al., 2023a). Moreover, TuMV-induced EVs are enriched in AtTET3, suggesting that TETs are involved in potyvirus infections (Movahed et al., 2019).

To date, the TET gene family has only been reported in *Arabidopsis* and rice (Wang et al., 2012a; Mani et al., 2015). Therefore, this study aimed to identify the TET gene family in sugarcane and determine its response to SCMV infection. This study provides valuable insights for the further exploration of TET functions and highlights the role of these proteins in the response to potyvirus infections.

## 2 Materials and methods

### 2.1 Plant materials and treatments

Tissue-cultured Xintaitang 22 (XTT22) plantlets were grown under a 14 h light/10 h dark cycle until reaching 15–25 cm in height with 4–5 fully expanded leaves and were individually inoculated with SCMV as previously described (Zhang H. et al., 2019; Yang et al., 2021). The plants were then inoculated with SCMV. XTT22 plantlets mock-inoculated with 0.01 M phosphate buffer (pH 7.0) served as negative controls. Sampling was conducted at 0 h, 12 h, 1 d, 3 d, 7 d, and 14 d, with three plantlets sampled at each time point. Roots, leaf rolls, +1 leaves, +7 leaves, +3 internodes, and +8 internodes were sampled from nine healthy 10-month-old XTT22 plants, which were divided into three groups of three plants each. *N. benthamiana* plants were cultured under a 16 h light/8 h dark cycle at 22°C and 60% humidity. All sampled plant materials were immediately frozen in liquid nitrogen and stored at −80°C.

### 2.2 Identification of putative sugarcane TETs

Genomic data of sugarcane cultivars XTT22 and R570 and *Saccharum* sp*ontaneum* AP85–441 were obtained from the Sugarcane Genome Database (https://sugarcane.gxu.edu.cn/scdb/download) (Zhang et al., 2018; Healey et al., 2024; Zhang et al., 2025c). Genomic data of *N. benthamiana* were obtained from an online website (http://lifenglab.hzau.edu.cn/Nicomics/Download/index.php) (Wang et al., 2024). Genomic data were obtained from an online website (https://phytozome-next.jgi.doe.gov/) (Goodstein et al., 2012). The Hidden Markov Model of TET (PF00335) was downloaded from an online database (http://pfam.xfam.org/) (Krogh et al., 2001) and used to query the genomes of *S.* sp*ontaneum* AP85-441, sugarcane cultivars XTT22 and R570, and *N. benthamiana*. The transmembrane domains of these TETs were analyzed on an online website (https://services.healthtech.dtu.dk/services/TMHMM-2.0/). The identified TETs were then verified using the CDD tool (https://www.ncbi.nlm.nih.gov/Structure/cdd/wrpsb.cgi).

### 2.3 Phylogenetic tree, physicochemical properties, and subcellular localization of sugarcane TETs

The BLASTp tool was used to screen the Phytozome v13 database (https://phytozome-next.jgi.doe.gov/) for TET proteins of maize (*Z. mays*), sorghum (*Sorghum bicolor*), millet (*Setaria italica*), wheat (*Triticum aestivum*), soybean (*G. max*), and potato (*S. tuberosum*), as well as the identified TETs in rice (Mani et al., 2015) or *Arabidopsis* (Wang et al., 2012a). The above TET sequences and identified TET sequences in *N. benthamiana* (NbTETs) or XTT22 (XTT22TETs) were subjected to multiple sequence alignment using MUSCLE v3.7, and a phylogenetic tree was constructed using the maximum likelihood method (bootstrap = 1,000) of MEGA X (Kumar et al., 2018). Then, the online software EvolView (https://evolgenius.info//evolview-v2) (https://evolgenius.info//evolview-v2) (He et al., 2016) was employed to refine the phylogenetic tree. The online website ExPASy (https://web.expasy.org/compute_pi/) was used to predict the physicochemical properties of sugarcane TET proteins. The subcellular localization of the identified TETs was predicted using the online website WoLF PSORT (https://wolfpsort.hgc.jp/) (Horton et al., 2007). Additionally, AlphaFold3 software (https://alphafoldserver.com/) was used to simulate the protein structure.

### 2.4 Conserved motifs and gene structure analysis

The conserved TET motifs in *S.* sp*ontaneum* AP85-441 (*SsTET*) and sugarcane cultivars R570 (*R570TET*) and XTT22 were obtained using MEME (https://web.mit.edu/meme/current/share/doc/overview.html) (Bailey et al., 2009). The parameters were set to search for 10 conserved motifs, with the remaining parameters set to default values. The gene-finding format 3 (gff3) files of the above species were downloaded from the Sugarcane Genome Database (https://sugarcane.gxu.edu.cn/scdb/download) (Zhang et al., 2018; Healey et al., 2024; Zhang et al., 2025c). The conserved motifs and gene structures of *SsTETs*, *R570TETs*, and *XTT22TETs* were visualized using TBtools 2.0.

### 2.5 *Cis*-acting elements analysis

The 2,000 bp sequences upstream of the coding sequence (CDS) region of *SsTETs*, *R570TETs*, and *XTT22TETs* were acquired from their corresponding genomic data. The *cis*-acting elements were predicted using PlantCARE software (https://bioinformatics.psb.ugent.be/webtools/plantcare/html/) and visualized using TBtools 2.0 (Chen et al., 2023).

### 2.6 Collinearity analysis

The collinearity and replication patterns of TETs were analyzed using MCScanX software (Wang et al., 2012b; Chen et al., 2023). Collinearity analysis of the genomes of sugarcane cultivars R570 and XTT22, sorghum (*S. bicolor*), maize (*Z. mays*), *Arabidopsis*, potato (*S. tuberosum*), millet (*S. italica*), and rice (*O. sativa*) was performed using TBtools 2.0 software. Genomic information of sorghum, maize, wheat, rice, *Arabidopsis*, and potato was downloaded from an online website (https://phytozome-next.jgi.doe.gov/) (Goodstein et al., 2012). In addition, the (Ka)/(Ks) value between homologous gene pairs was calculated based on the correlation of homology using TBtools 2.0.

### 2.7 Transcriptomic data analysis

The transcriptome data of AP85–441 and XTT22 are available in an online repository (https://sugarcane.gxu.edu.cn/scdb/download) (Hu et al., 2018; Zhang et al., 2025c). These data were collected at the seedling stage (35 d), early maturity stage (270 d), and mature stage (360 d). RNA-seq data from the leaves at four different developmental stages were collected to investigate the expression profiles of the TET family. The transcription fragments per million bases (FPKMs) of *SsTETs* or *XTT22TET* were used to generate heat maps and conduct cluster analysis using TBtools 2.0 (Chen et al., 2023).

### 2.8 RNA isolation, cDNA synthesis, and RT-qPCR

Total RNA was extracted from SCMV-infected sugarcane plants and healthy sugarcane plants using the TRIzol method. The PrimeScript^®^ RT-PCR kit (TaKaRa Biotechnology Co., Ltd., Dalian, China) was used to synthesize the first-strand cDNA. Special primers (**Supplementary Table S1**) were designed to quantify the TET genes by RT-qPCR with *eEF-1a* and *Actin* used as internal references (Iskandar et al., 2004; Ling et al., 2014; Xue et al., 2014). The relative expression levels of *TET* genes were analyzed using the 2^−ΔΔCt^ method. All primers used for RT-qPCR are listed in **Supplementary Table S1**.

### 2.9 Plasmid construction

Special primers (**Supplementary Table S1**) were designed to construct the plasmids. For the yeast two-hybrid (Y2H) experiments, DNA fragments and Y2H vectors were ligated individually at the *Ecor* I and *Sam* I sites. The bait vectors of TuMV-6K2 and SCMV-6K2 are from our previous work (Zhang H. et al., 2019; Zhang et al., 2024), and the target genes were cloned into the prey vector pPR3-N. In addition, three *TET* genes cloned from XTT22 (*ScTETs*) were inserted into the pBT-STE vector to investigate the interactions among ScTETs. Gateway technology was employed to construct the plasmids for the bimolecular fluorescence complementation (BiFC) assays. The 6K2-YN vector was generated in a previous research study (Zhang et al., 2024). For the subcellular localization experiments, all DNA fragments were inserted into the vectors via the *Kpn* I and *Sal* I sites. For the luciferase complementation assays (LCAs), DNA fragments and LCA vectors were ligated at the *Kpn* I and *Sal* I sites. All plasmids constructed in this study were verified through sequencing.

### 2.10 Y2H, BiFC, LCA, and subcellular localization assays

For the Y2H assays, paired prey and bait vectors were co-transformed into the yeast strain *NMY51*. Then the transformed yeast cells were spread onto the double dropout medium (DDO) SD/-Trp/-Leu solid medium and cultured at 30°C for 48–72 h. Yeast single colony grown on DDO solid medium were suspended in DDO liquid medium to OD_600_ = 0.6. Ten-fold serial dilutions of yeast were spotted onto DDO or quadruple dropout medium (QDO) SD/-Trp/-Leu/-His/-Ade solid medium and cultured at 30°C for 48–72 h. The yeast cells co-transformed with pNubG-Fe65 and pTSU2-APP served as positive controls, while those co-transformed with pNubG-Fe65 and pPR3-N served as negative controls, as previous report (Zhang H. et al., 2019).

For the BiFC experiments, complementary vectors containing the target genes for the identification of interactions were co-transformed into *Agrobacterium tumefaciens* GV3101 and cultured to an OD_600_ of 0.2. Equal volumes of each culture were mixed and infiltrated into *N. benthamiana* leaves using a needleless syringe. *Agrobacterium*-infiltrated plants were grown under normal conditions for 48–72 h (Yang et al., 2021).

For the LCA assays, the target genes were cloned into the pCAMBIA1300-nLUC and pCAMBIA1300-cLUC vectors. Subsequently, these recombinant plasmids were introduced into different regions of the same *N. benthamiana* leaf via *A. tumefaciens* infiltration, with the final concentration of *A. tumefacien*s set at an OD_600_ of 0.4. Thereafter, a 0.2 mM luciferase substrate was infiltrated into the same regions, and imaging was performed 2 d post-infiltration (dpi) using a low-light-cooled CCD imaging system (Amersham Imager 680, GE, USA).

For the subcellular localization experiments, complementary vectors containing the target genes for the identification of interactions were transformed into *A. tumefaciens* GV3101 and cultured to an OD_600_ of 0.2. Equal volumes of each culture were mixed and agroinfiltrated into *N. benthamiana* leaves using a needleless syringe. AtCDPK9-mCherry was used as a PM marker (Cheng et al., 2017). *Agrobacterium*-infiltrated plants were grown under normal conditions for 48–72 h.

### 2.11 Confocal microscopy

Images were digitally acquired using a Leica SP8X confocal microscope (Leica, Wetzlar, Germany). Yellow fluorescent protein (YFP) was excited at 514 nm, and the emitted light was captured at 530–590 nm. The excitation wavelength of mCherry was 587 nm, and the collection wavelength was 610 nm. The excitation and emission wavelengths of green fluorescent protein (GFP) were 514 and 530–590 nm, respectively. Images were analyzed using Leica Microsystems.

## 3 Results

### 3.1 Identification and phylogenetic analysis of the TET gene family

**Figure 1:**
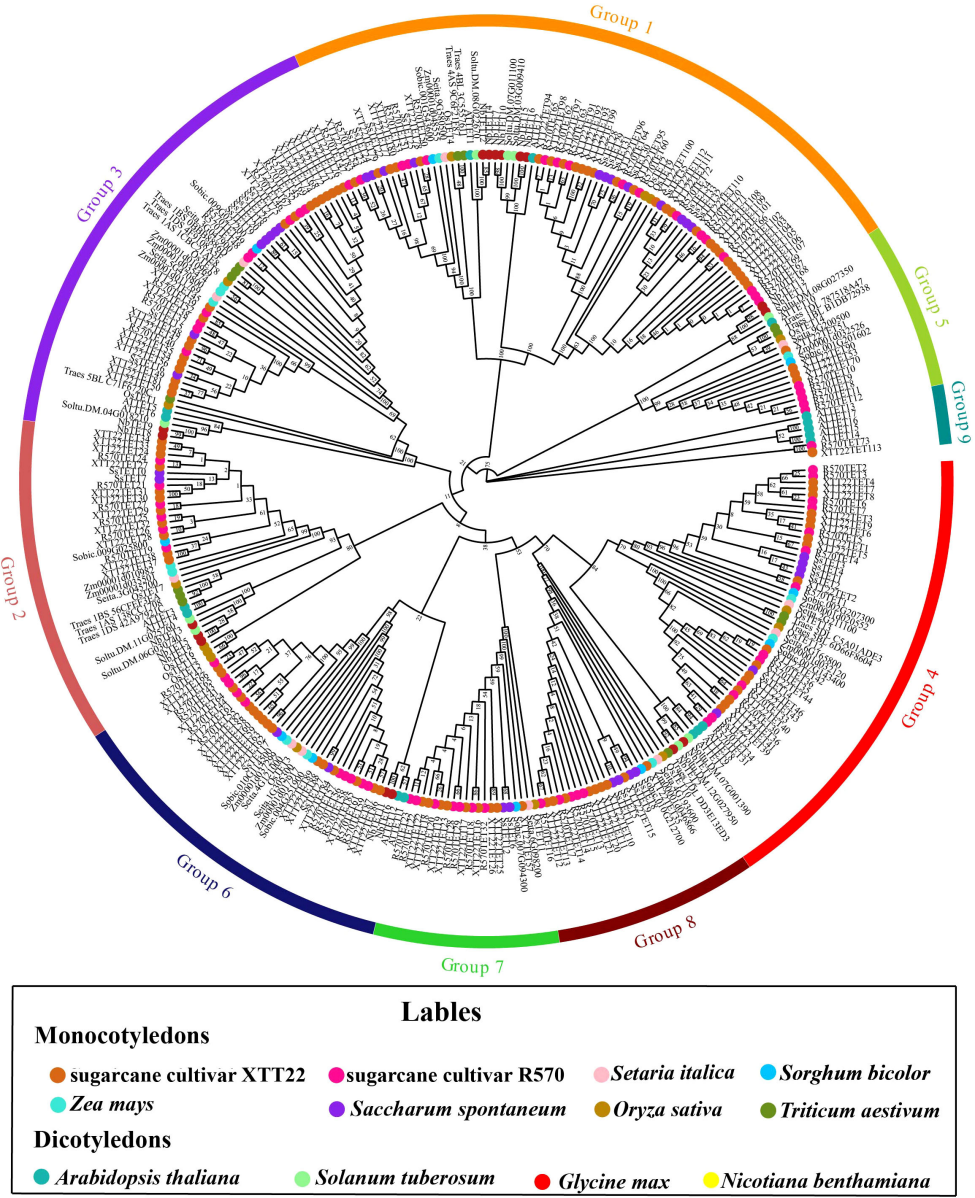
Phylogenetic tree analysis of Tetraspanins (TETs) of *Saccharum* species and other plant species. The phylogenetic tree was constructed using the maximum likelihood method with 1,000 bootstrap replicates. The TETs sequences are from the databases of 8 monocotyledons (sugarcane cultivar R570, sugarcane cultivar XTT22, *Oryza sativa*, *Zea mays*, *Setaria italic*, *S.* sp*ontaneum*, *Triticum aestivum* and S*orghum bicolor*) and 4 dicotyledons (*Arabidopsis thaliana*. *Nicotiana benthamiana*, *Glycine max*, *Solanum tuberosum*). These TETs were grouped into 9 distinct groups and annotated with different colors.

We identified 35, 73, 113, and 17 members of the TET gene family in *S.* sp*ontaneum* AP85-441, sugarcane cultivar R570, sugarcane cultivar XTT22, and *N. benthamiana*, respectively (**Supplementary Table S2**). Phylogenetic analysis indicated that the TETs from sugarcane cultivars XTT22 and R570 were clustered into nine evolutionary groups, whereas none of SsTETs from *S.* sp*ontaneum* were distributed in Groups 5 and 9. Groups 7 and 8 contained only the TETs from monocotyledonous plants (**Figure 1**). The TETs of monocotyledonous and dicotyledonous plants were further clustered into different subgroups within the same group. AtTETs were clustered into seven groups, that is, Groups 1, 2, 3, 4, 5, 6, and 9, which is consistent with a previous report (Wang et al., 2012a). Surprisingly, ScTSPAN18, previously identified as a protein interacting with SCMV-6K2, was not included in the TET gene family (Zhang H. et al., 2019). To investigate the differences between ScTSPAN18 and the typical TETs AtTET1 and OsTET1, AlphaFold3 was used to simulate the protein structures. Notably, the LEL of ScTSPAN18 was small, and the conserved motifs were absent compared with typical TETs (**Supplementary Figure S1**).

### 3.2 Conserved motifs, gene structure, and physicochemical properties of TETs

Ten conserved motifs, distributed among Groups 1–8 in the same distribution order, were identified in the SsTETs, R570TETs, and XTT22TETs. For the TETs in Group 9, only motifs 1, 2, 5, 7, and 8 were detected (**Supplementary Figure S2A**). Gene structure analysis revealed that the ETs in Groups 1–4 and 6–9 contained 2–4 exons (**Supplementary Figure S2B**), whereas those in Group 5 contained more than 10 exons (**Supplementary Figure S2B**).

Physicochemical analysis of the sugarcane TET proteins revealed amino acid lengths ranging from 205 to 853, theoretical isoelectric points between 7.75 and 9.91, and instability coefficients between 29.53 and 58.03. Subcellular localization of the above proteins was predicted using a protein subcellular localization website (**Supplementary Table S3**). Most of the TETs in sugarcane were localized in the PM, although some were localized in the Golgi apparatus and ER (**Supplementary Table S3**).

### 3.3 Collinearity and chromosomal localization of the *TET* gene

**Figure 2:**
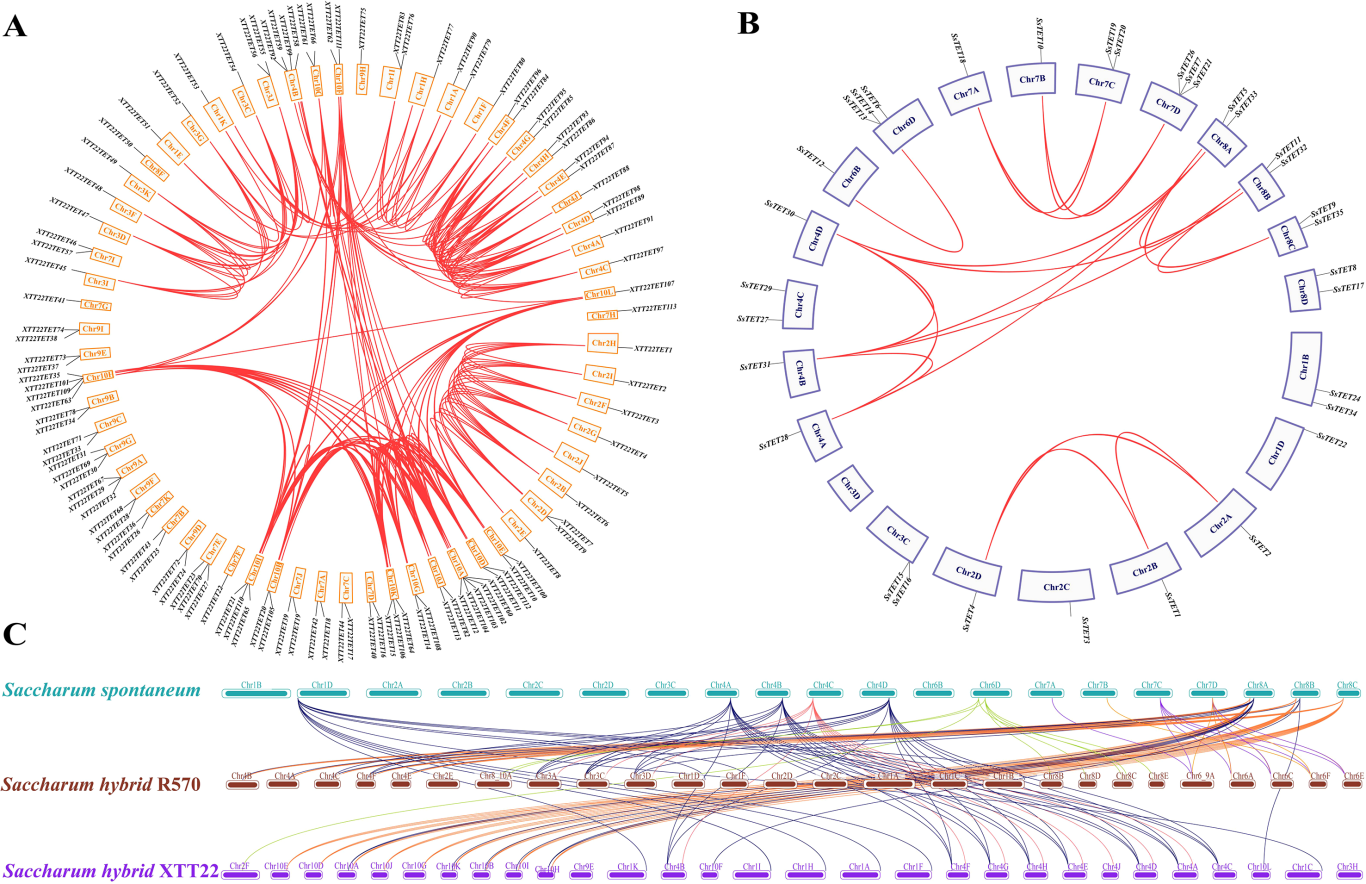
Interchromosomal and intrachromosomal collinearity relationship analysis of the *TET* gene families in *Saccharum* species. **(A)** Intrachromosomal collinearity analysis of the TET gene family in sugarcane cultivar XTT22. Red lines indicate duplicated *TET* gene pairs. **(B)** Intrachromosomal collinearity analysis of the TET gene family in *S.* sp*ontaneum*. Red lines indicate duplicated *TET* gene pairs. **(C)** Interchromosomal collinearity analysis of the TET gene families among in *S.* sp*ontaneum*, sugarcane cultivar R570, XTT22. The lines represent the TET homologous gene pairs.

**Figure 3:**
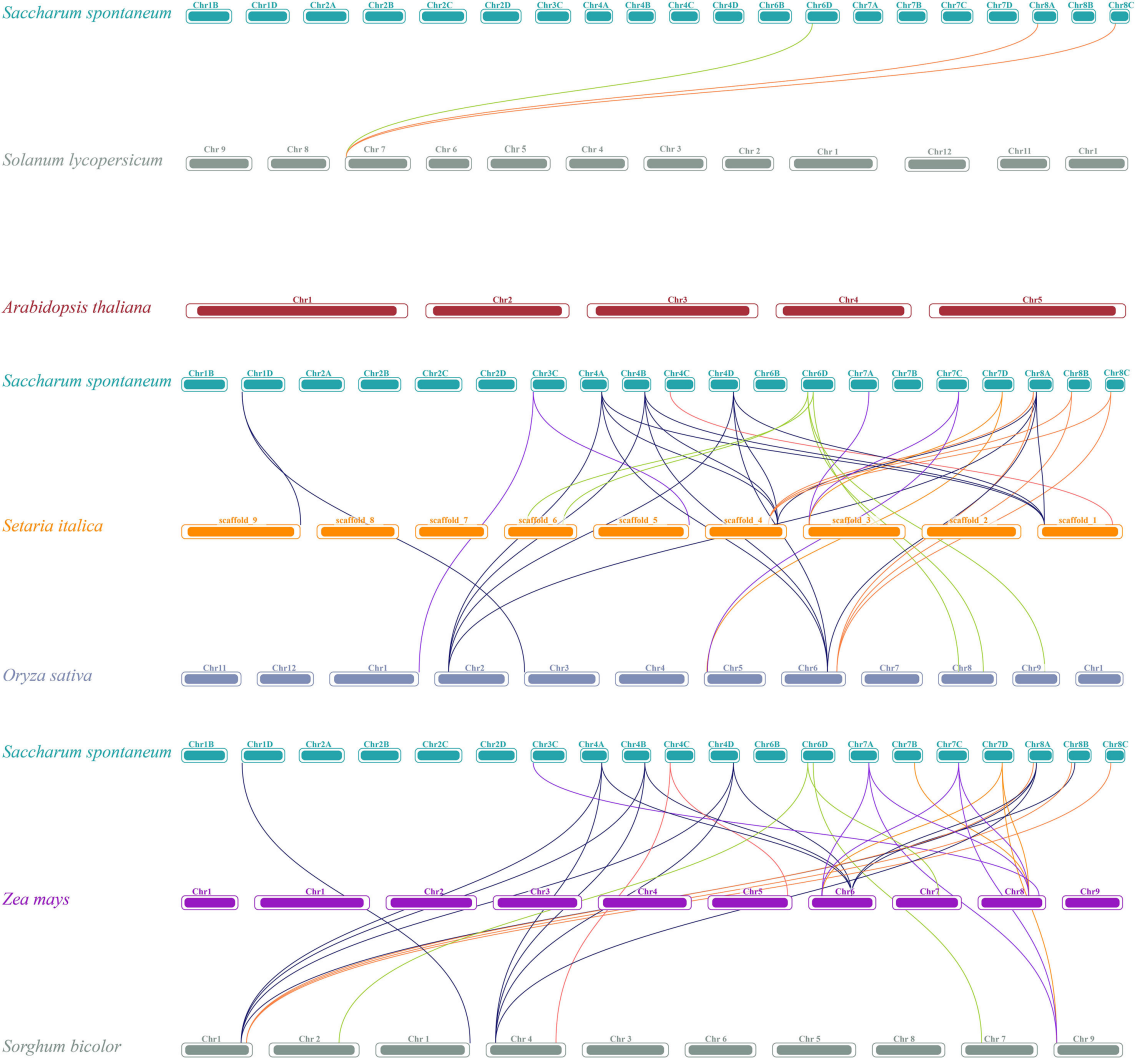
Synteny analysis of *TET* genes between *S.* sp*ontaneum* and other plant species (*O. sativa*, Z*. may*, *S. italic*, *S. bicolor*, *A. thaliana*, and *S. Lycopersicum*). The lines represent the *TET* homologous gene pairs.

To understand the evolution of the TET gene family, the intra- and inter-species collinearity of TET genes in XTT22, R570, and *S.* sp*ontanerum* AP85–441 was investigated. Intraspecies collinearity analysis revealed 266, 20, and 260 pairs of collinear TETs in XTT22 (**Figure 2A**), *S.* sp*ontanerum* AP85-441 (**Figure 2B**), and R570 (**Supplementary Figure S3**), respectively. Interspecies collinearity analysis revealed 96 and 86 pairs of collinear TETs between AP85–441 and R570 and XTT22, respectively (**Figure 2C**). To gain more evolutionary information of the TET gene family, we analyzed the synteny of *TET* genes from *S.* sp*ontaneum* with those from *Setaria italica*, sorghum, maize, rice, *Arabidopsis*, and *Solanum lycopersicumt*. The results showed that there are 19 pairs between *Setaria italica* and *S.* sp*ontaneum*, 19 pairs of homologous genes between *sorghum* and *S.* sp*ontaneum*, 15 pairs between *maize* and *S.* sp*ontaneum*, 18 pairs between rice and *S.* sp*ontaneum*, 3 pairs between *Solanum lycopersicumt* and *S.* sp*ontaneum* and no pairs between *Arabidopsis* and *S.* sp*ontaneum* (**Figure 3**). MCScanX analysis showed that whole-genome or segmental duplication was the primary origin of TETs in AP85-441 (45.7%), XTT22 (61.0%), and R570 (84.9%) (**Supplementary Table S4**). The Ka/Ks ratios of all the TET gene pairs were <1 (**Supplementary Table S5**), suggesting that homologous genes among rice, sorghum, *S. italica*, maize, and *S.* sp*ontaneum*, have undergone strong purifying selection.

Chromosomal mapping demonstrated uneven distribution of the *TET* genes in XTT22, with most chromosomes containing 1–2 and a few containing 3–5 genes (**Supplementary Figure S4**; **Supplementary Table S6**). The chromosomal distribution of *TETs* in AP85-441 (**Supplementary Figure S5**) and R570 (**Supplementary Figure S6**) was similar to that in XTT22 (**Supplementary Table S6**).

### 3.4 *Cis*-acting elements in *TET* genes

In total, 20 *cis-*acting elements were predicted in the promoter regions of the *TETs* (**Supplementary Figure S7**; **Supplementary Table S7**). ARE (94.6%), G-box (93.7%), ABRE (91.4%), TGACG-motif (90.0%), and CGTCA-motif (89.4%) were distributed in the promoter regions of more than 80% of the *TETs*, indicating their extensive involvement in responses to stress, light, and hormones. GATA box was only predicted in Group 3. In Group 6, the upstream region of the *TET* gene did not contain a CCAAT box, GC motif, or GA motif. In Group 8, the upstream region of the *TET* gene did not contain the GCN4 motif or GC motif (**Supplementary Figure S7**).

### 3.5 Expression patterns of *TETs* based on the transcriptomic data

**Figure 4:**
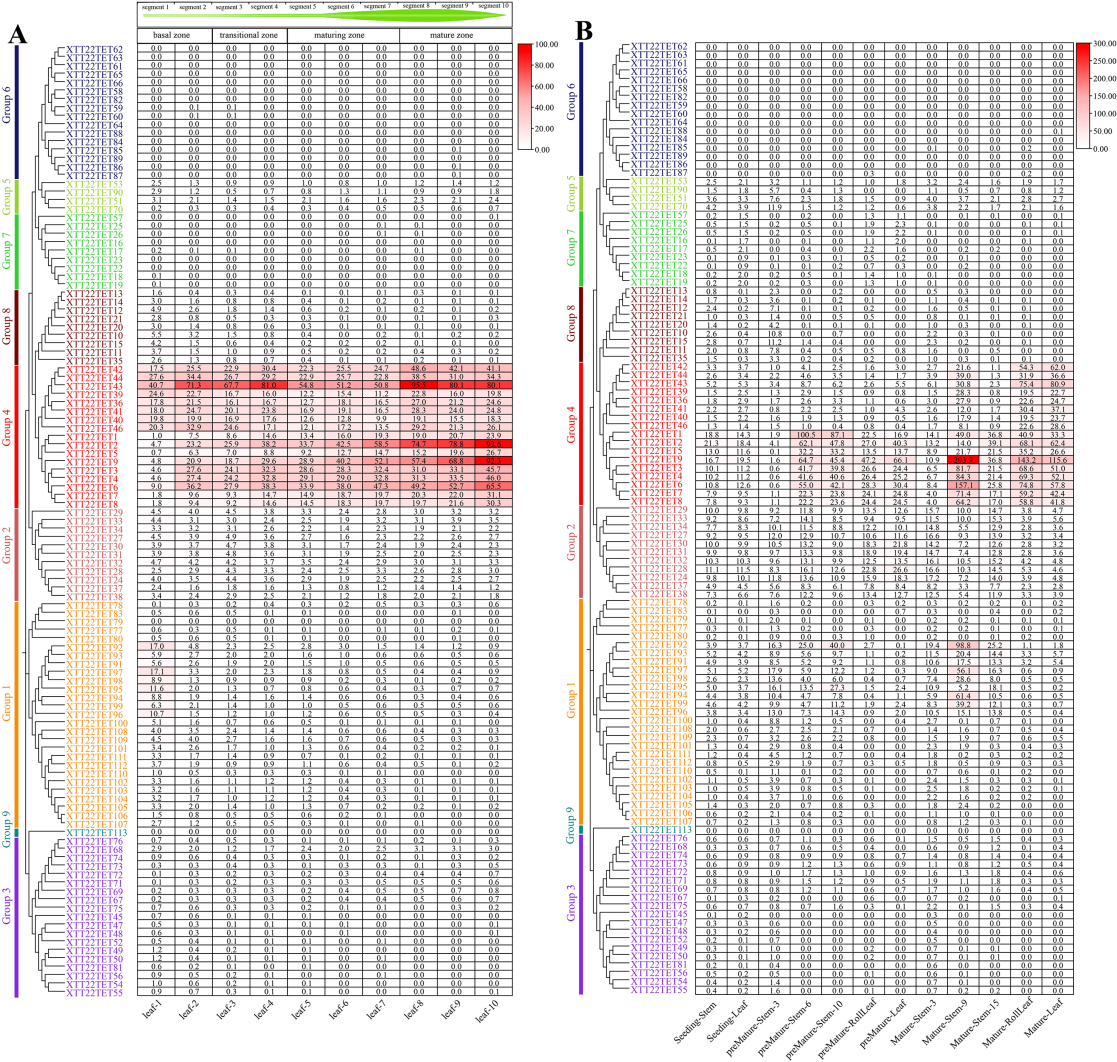
The expression patterns of *TET* genes in different tissues and across leaf gradients in sugarcane cultivar XTT22. **(A)** The expression patterns of *XTT22TET* genes based on FPKM across leaf gradients in sugarcane cultivar XTT22. **(B)** The expression patterns of *XTT22TET* gene family based on FPKM in different tissues of different stages in sugarcane cultivar XTT22.

Transcriptomic analysis of the continuous developmental gradient of leaves of XTT22 and *S.* sp*ontaneum* AP85–441 was performed to investigate the potential functions of *TET* genes in photosynthesis. Group 4 *XTT22TETs* were highly expressed in the mature zone, whereas Group 1 *XTT22TETs* were highly expressed in the transitional zone, with other *XTT22TET* genes exhibiting low or no expression (**Figure 4A**; **Supplementary Table S8**). For the *SsTETs* in the leaves of *S.* sp*ontaneum* AP85-441, Group 4 *SsTETs* were highly expressed in the transition and maturation zones. Groups 1, 2, and 8 *SsTETs* were expressed in the basal zone, whereas Group 3 *SsTETs* exhibited low expression in the leaves (**Supplementary Figure S8A**; **Supplementary Table S8**).

The transcriptomic analysis of different tissues across different developmental stages of XTT22 revealed that Group 4 *TETs* were highly expressed in mature leaves and internodes (**Figure 4B**). Members of Groups 2 and 8 were expressed in different tissues in the seedling and premature stages, whereas members of Groups 3, 5, and 7 exhibited consistently low expression across all developmental stages, and members of Groups 6 and 9 were not expressed (**Figure 4B**; **Supplementary Table S9**). For *S.* sp*ontaneum* AP85-441, Group 4 *SsTETs* were highly expressed in the stems and leaves in the premature and mature stages, and four *SsTETs* in Group 1 showed relatively high expression in premature and mature stems (**Supplementary Figure S8B**; **Supplementary Table S9**).

### 3.6 Cloning and characterization of ScTETs

**Figure 5:**
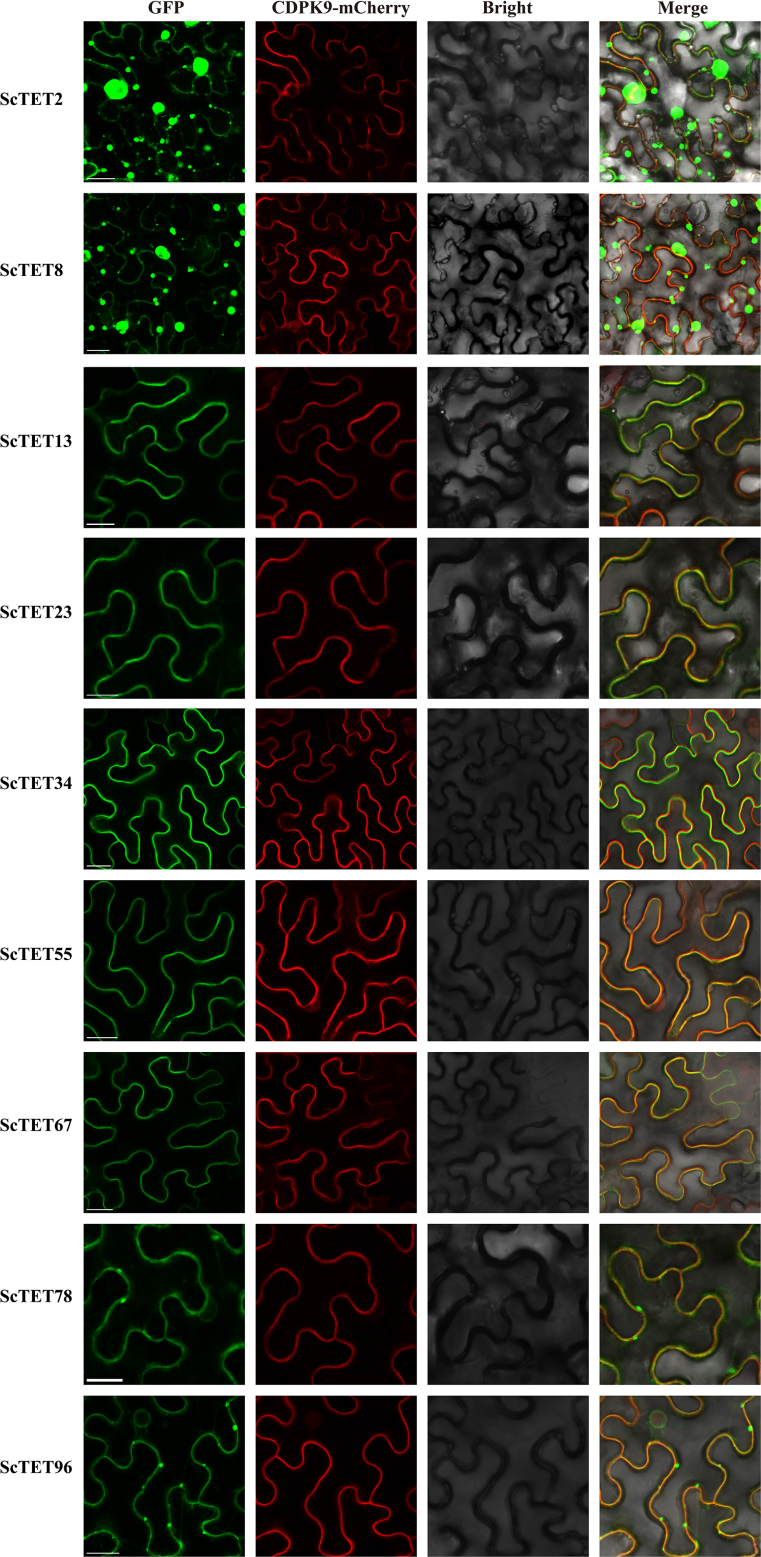
Subcellular localization of ScTETs. Agrobacteria harboring GFP fusion proteins were individually agroinfiltrated into *N. benthamiana* leaves. The images were captured at 48 h post infiltration. The plasma membrane was indicated using AtCDPK9-mCherry, Bar=20 μm.

The results of bioinformatics and transcriptome data analyses were used to clone the following nine genes from Groups 1, 2, 3, 4, 7, and 8 of XTT22: *XTT22TET2*, *XTT22TET8*, *XTT22TET13*, *XTT22TET23*, *XTT22TET34*, *XTT22TET55*, *XTT22TET67*, *XTT22TET78*, and *XTT22TET96*, which were named *ScTET2*, *ScTET8*, *ScTET13*, *ScTET23*, *ScTET34*, *ScTET55*, *ScTET67*, *ScTET78*, and *ScTET96*, respectively (**Supplementary Table S2**). Sequence alignment and phylogenetic tree analysis revealed 98.53% similarity between ScTET8 and XTT22TET8 and 98.94% similarity between ScTET55 and *XTT22TET55*. The similarity between the other seven ScTETs was identical to the corresponding identified XTT22TETs. Subcellular localization assays showed that the fluorescence signals of ScTET13-GFP, ScTET23-GFP, ScTET34-GFP, ScTET55-GFP, ScTET67-GFP, ScTET78-GFP, and ScTET96-GFP overlapped with the fluorescence signal of AtCDPK9-mCherry (**Figure 5**), indicating that they were mainly located on the PM, aligning with the subcellular localization prediction results (**Supplementary Table S3**). The ScTET78-GFP and ScTET96-GFP exhibited punctate structures on the PM (**Figure 5**), whereas the ScTET2-GFP and ScTET8-GFP formed spherical structures of varying sizes ranging from 0.5 to 20 μm in diameter within the cells (**Figure 5**; **Supplementary Figure S9**).

**Figure 6:**
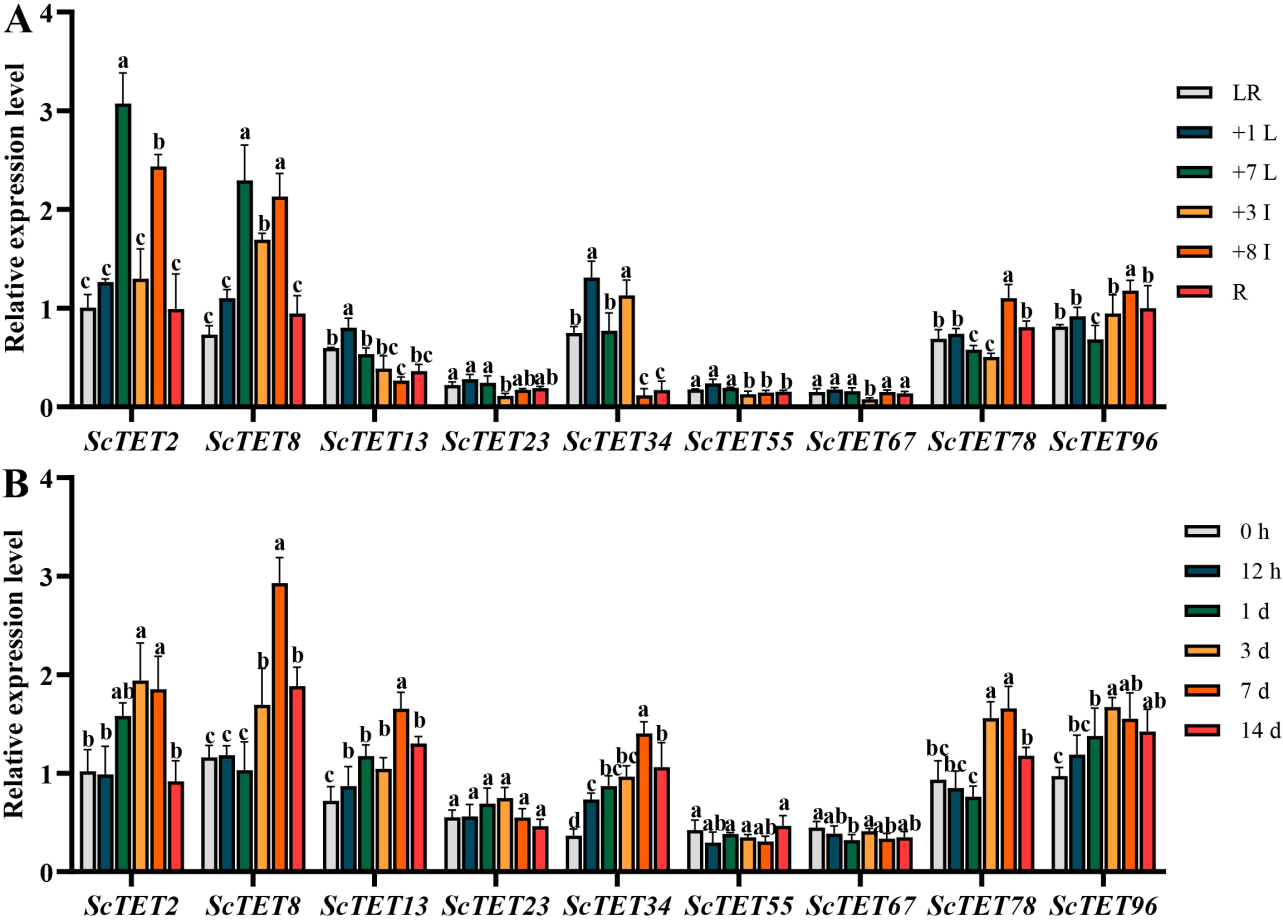
The expression profiles of nine *ScTETs* in different tissues of sugarcane cultivar XTT22, or under the challenge of sugarcane mosaic virus (SCMV). **(A)** The expression profiles of nine *ScTETs* in different tissues of sugarcane cultivar XTT22. LR: leaf roll; +1 L: the 1st leaf; +7 L: the 7st leaf; +3 I: the 3rd internode; +8 I: the 8th internode and R: root. **(B)** The expression profiles of nine *ScTETs* in the leaves of sugarcane cultivar XTT22 under the challenge of SCMV. Plants mock inoculated with 0.01 mM phosphate buffer (pH 7.0) were used as the negative controls. The Y axes indicates the relative expression levels of *ScTETs*. The X axes indicates the time point of materials collection. Error bars indicate SD (*n* = 3), a, b, c and e indicate significance at the corresponding time points, Student’s *t*-test, *P* < 0.05. Results were representative of three independent experiments.

The expression patterns of *ScTETs* in different tissues of the sugarcane cultivar XTT22 were analyzed via RT-qPCR. *ScTET2*, *ScTET8*, *ScTET34*, *ScTET78*, and *ScTET96* exhibited significantly higher expression than the other genes (**Figure 6A**). *ScTET2* and *ScTET8* were highly expressed in the +7 leaf and +8 internode, *ScTET34* was highly expressed in the +1 leaf and +3 internode, and *ScTET78* and *ScTET96* were highly expressed in the +8 internode (**Figure 6A**).

All *ScTETs* except *ScTET23* were differentially expressed upon SCMV infection (**Figure 6B**). Expressions of *ScTET2*, *ScTET8*, *ScTET13*, *ScTET34, ScTET78*, and *ScTET96* increased significantly at 3 dpi and peaked 7 dpi (**Figure 6B**). All genes except *ScTET96* were downregulated at day 14 (**Figure 6B**).

### 3.7 Interaction between ScTETs and SCMV-6K2

**Figure 7:**
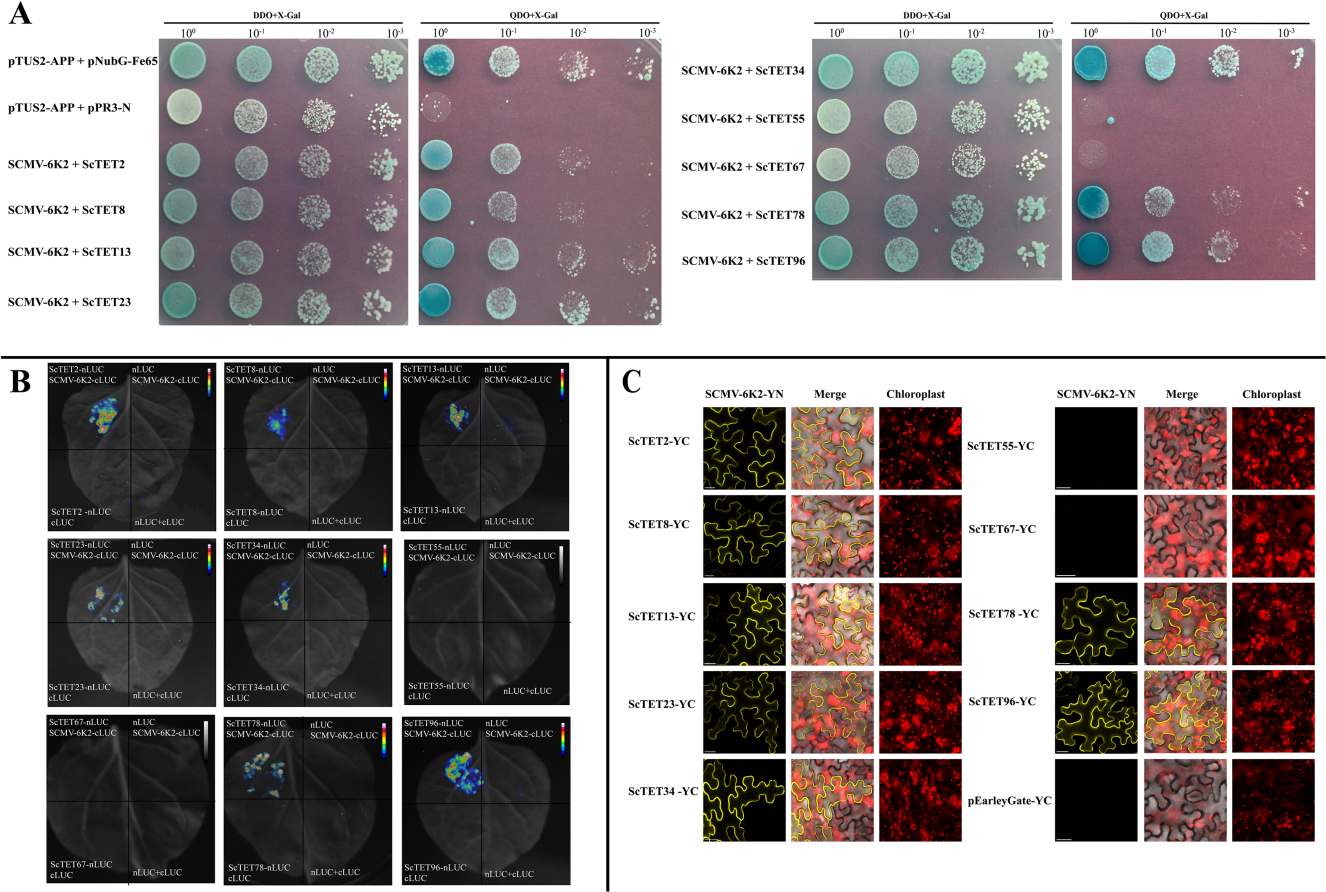
Interaction of 9 ScTETs with SCMV-6K2 by BiFC, Y2H and Luc assays. **(A)** Y2H assays. pPR3-ScTETs were individually pairwise co-transformed with the vector pBT-STE-SCMV-6K2 into the yeast NYM51 cells in a 10×dilution series of 10-μL aliquots which were then plated on a non-selective medium, SD/-Leu/-Trp or quadruple dropout medium, SD/-Leu/-Trp/-His/-Ade supplemented with X-Gal. Yeast cells co-transformed with pTUS2-APP and pNubG-Fe65 were used as a positive control. pTUS2-APP and pPR3-N were used as negative controls. **(B)** Luc assays. Agrobacteria harboring nLuc/cLuc fusion proteins were individually pairwise co-infiltrated into *N. benthamiana* leaves. The leaf epidermal cells pairwise co-transformed with ScTETs-nLUC and cLUC, cLuc-6K2 and nLuc, cLuc and nLuc were used as negative controls. **(C)** BiFC assays. Agrobacteria harboring YC/YN fusion proteins were individually pairwise co-infiltrated into *N. benthamiana* leaves. The images were captured at 48 h post infiltration. YC and SCMV-6K2-YN were used as negative controls. Bar=20 μm.

The interactions of the nine ScTETs with SCMV-6K2 were analyzed using Y2H, BiFC, and LCA assays. For the Y2H assays, pTUS2-APP- and pNUbG-Fe65-co-transformed NYM51 served as the positive controls, and pTUS2-APP- and pPR3-N-co-transformed NYM51 served as the negative controls. pPPR3-ScTET2, pPPR3-ScTET8, pPPR3-ScTET13, pPPR3-ScTET23, pPPR3-ScTET34, pPPR3-ScTET55, pPPR3-ScTET67, pPPR3-ScTET78, and pPPR3-ScTET96 were co-transferred with pBT-STE-SCMV-6K2 into the yeast NYM51. In DDO and QDO media containing X-gal, the yeast cells harboring pPPR3-ScTET2, pPPR3-ScTET8, pPPR3-ScTET13, pPPR3-ScTET23, pPPR3-ScTET34, pPPR3-ScTET78, or pPPR3-ScTET96 with pBT-STE-SCMV-6K2 grew normally as the positive controls (**Figure 7A**). In contrast, the yeast cells harboring pPPR3-ScTET55 or pPPR3-ScTET67 with pBT-STE-SCMV-6K2 grew only on DDO medium but not QDO (**Figure 7A**). These results demonstrated that all nine ScTETs, except ScTET55 and ScTET67, interacted with SCMV-6K2.

LCA (**Figure 7B**) and BiFC assays (**Figure 7C**) yielded results similar to those of the Y2H assays. Interestingly, the BiFC assays showed that all interactions occurred at the PM. These interactions resulted in the loss of intracellular localization and spherical structures of ScTET2 and ScTET8, as well as the disappearance of the punctate PM-associated structures formed by ScTET78 and ScTET96 (**Figure 7C**).

### 3.8 Interactions and self-interactions among ScTETs

**Figure 8:**
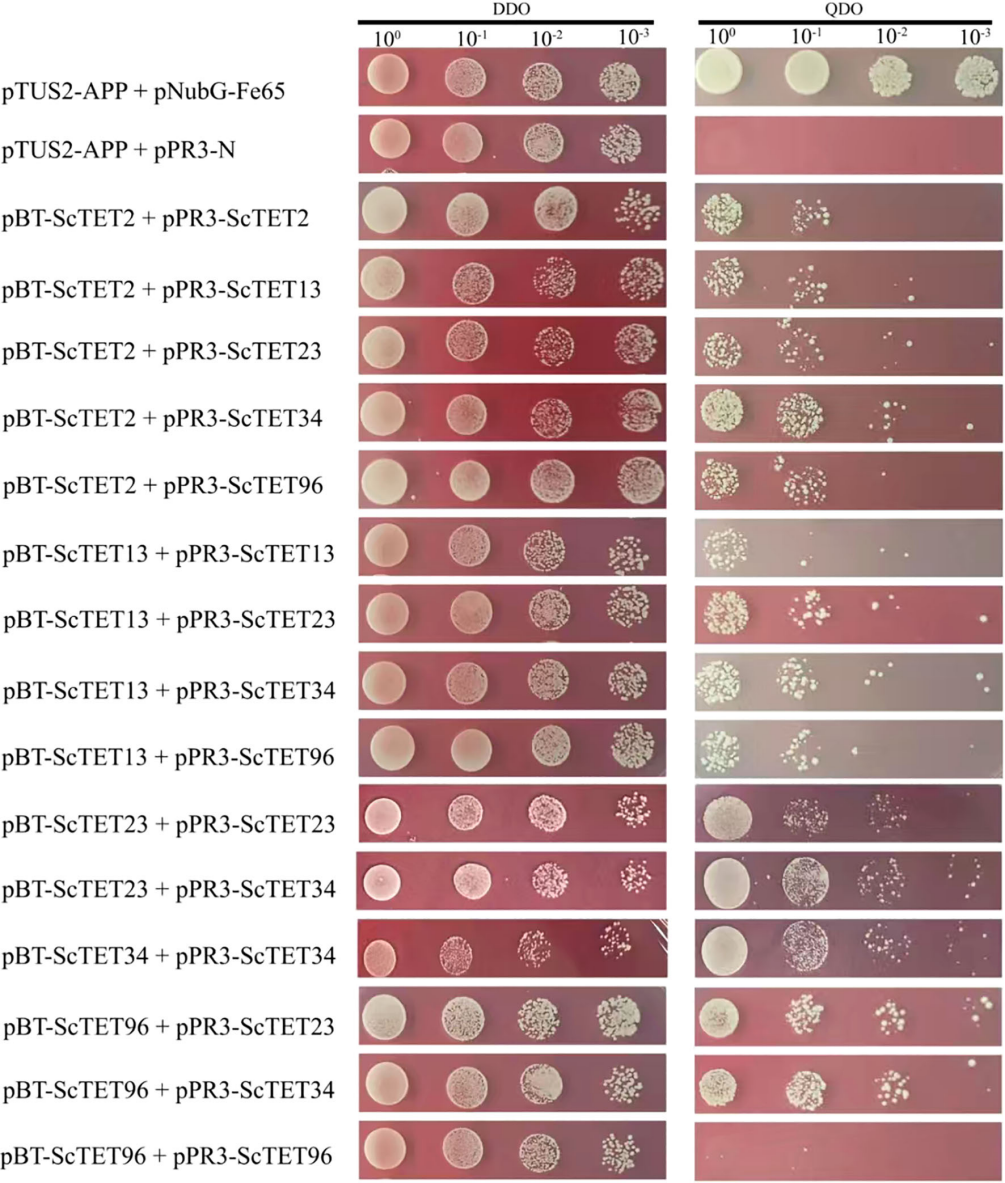
Self-interaction of ScTETs by Y2H assays. Combinations of pBT-STE-ScTET2 plus pPR3-ScTET2, pBT-STE-ScTET13 plus pPR3-ScTET13 and pBT-STE-ScTET96 plus pPR3-ScTET96 were individually transformed into the yeast NYM51. Yeast cells co-transformed with pTUS2-APP and pNubG-Fe65 were used as a positive control, while yeast cells co-transformed with pTUS2-APP and pPR3-N were used as negative controls.

Interactions and self-interactions among the TET members contribute to TEM formation (Charrin et al., 2002; Jimenez-Jimenez et al., 2019; Huang et al., 2022). We conducted Y2H assays to investigate the possible interactions and self-interactions among the nine ScTETs and observed extensive interactions among the ScTETs (**Figure 8**). ScTET2, ScTET13, ScTET23 and ScTET34 interacted with other ScTETs and could also self-interact. ScTET96 also interacted with other ScTETs but could not self-interact (**Figure 8**).

### 3.9 Interaction of AtTETs or NbTETs with TuMV-6K2 assessed using Y2H

The intercellular movement of viruses is a key step in establishing systemic infection. AtTET3 is localized in the plasmodesmata and required for CMV to establish systemic infection (Fernandez-Calvino et al., 2011; Zhu et al., 2022). To investigate whether TET is widely involved in the interaction with potyvirus-6K2, we cloned four *AtTET* genes, *AtTET1*, *AtTET3*, *AtTET7*, and *AtTET8*, which are localized in the plasmodesmata of *Arabidopsis* (Fernandez-Calvino et al., 2011; Boavida et al., 2013). In addition, we cloned the 17 *NbTET* genes identified in this study (**Supplementary Table S2**). These genes were individually inserted into the pPR3-N vector and then individually co-transformed with pBT-STE-TuMV-6K2 into yeast NMY51. Notably, all these TETs interacted with TuMV-6K2 (**Supplementary Figures S10**, **S11**).

## 4 Discussion

Genome-wide identification of the TET gene family has only been previously performed in *Arabidopsis* and rice, which identified 17 and 15 TETs, respectively (Wang et al., 2012a; Mani et al., 2015). In the present study, we identified 35, 113, 73, and 17 TETs in *S.* sp*ontaneum* AP85-441, sugarcane cultivars XTT22 and R570, and *N. benthamiana*, respectively. We achieved the Phytozome database and screened 11, 10, 12, 14, 9, and 20 TETs for millet, sorghum, maize, wheat, potato, and soybean, respectively. The number of TETs in *Saccharum* species was much higher than that in other plant species, likely because of the highly polyploid characteristics of sugarcane (Zhang et al., 2022; Healey et al., 2024; Zhang et al., 2025c). XTT22TET and R570TET were clustered in nine phylogenetic groups, whereas SsTETs were absent in Groups 5 and 9 (**Figure 1**). Additionally, the *TET* genes in Group 5 contained a significantly higher number of exons at up to 10 or more compared with the other eight groups (**Figure 1B**). This feature is considered characteristic of the original *TET* genes (Garcia-España et al., 2008), as demonstrated by *AtTET10* and *OsTET14* (Mani et al., 2015). *S.* sp*ontanerum* and *S. officinarum* are recognized as the main progenitors of modern sugarcane cultivars, and *S. officinarum* is derived from *S. robustum* (Zhang J. et al., 2019; Zhang et al., 2022). We speculated that sugarcane TET genes in Groups 5 and 9 may have originated from *S. robustum*. Additionally, as only monocotyledonous plant TETs were present in Groups 7 and 8, we speculated that these groups are unique to monocotyledons (**Figure 1**), which is consistent with a previous report (Wang et al., 2012a). Interestingly, GATA-box is only present in the promoter region of Group 3 *TETs* (**Supplementary Figure S7**). As GATA-box usually responds to light signaling, we speculate that Group 3 *TETs* are involved in photosynthesis (Gangappa and Chattopadhyay, 2013). Expansion of the TET gene family in *Saccharum* species appears to have primarily occurred through WGD/fragmentation and dispersed replication (**Supplementary Table S4**), which is in line with previous reports (Zhang et al., 2018; Li et al., 2021; Wang et al., 2023). ScTSPAN18, which was identified in our previous study (Zhang H. et al., 2019), did not belong to the TET family (**Supplementary Figure S1**). In early studies on animal TETs, the L6D protein was mistakenly classified into the TET family (Wright et al., 2000; Boucheix and Rubinstein, 2001). Structural simulation using AlphaFold3 showed that the LEL of L6D lacked conserved motifs (**Supplementary Figure S1**). Homologous proteins with similar structural features were identified in *Arabidopsis* (four), rice (one), sorghum (two), and maize (three) using the Phytozome database. However, the biological functions of these proteins require further investigation.

Transcriptomic data analysis revealed that the 119 *XTT22TET* genes identified in this study were differentially expressed across various tissues and developmental stages (**Figure 4**; **Supplementary Figure S8**), indicating that they are extensively involved in sugarcane growth and development, which is consistent with the findings in *Arabidopsis* (Boavida et al., 2013). Plant viruses have undergone long-term co-evolution with their hosts and cannot establish systemic infections without interacting with host factors (Benitez-Alfonso et al., 2010; Niehl and Heinlein, 2011). Nine *ScTET* genes were cloned from the sugarcane cultivar XTT22, and RT-qPCR revealed their differential expression following SCMV infection (**Figure 6**), indicating that they all responded to the SCMV infection. Protein–protein interaction assays revealed interactions between all ScTETs and SCMV-6K2, except for ScTET55 and ScTET67 in Group 3 (**Figure 7**). Plasmodesmata are important channels for the intercellular movement of plant viruses (Valli et al., 2007; Chung et al., 2008; Wei et al., 2010; Cheng et al., 2017; Chai et al., 2020; Cheng et al., 2020; Xiao et al., 2022; Hýsková et al., 2024). Four plasmodesmata-localized AtTETs—AtTET1 (Group 1), AtTET3 (Group 2), AtTET7, and AtTET8 (Group 4) (Fernandez-Calvino et al., 2011; Boavida et al., 2013; Johnston et al., 2023)—interacted with TuMV-6K2 (**Supplementary Figure S10**). AtTET3 is essential for the intercellular movement of the CMV (Zhu et al., 2022). Based on this, we hypothesized that TETs may also play a role in the intercellular movement of potyviruses. Specifically, ScTET78 and ScTET96 (Group 1), ScTET34 (Group 2), and ScTET2 and ScTET8 (Group 4), which belong to the same phylogenetic groups as the AtTETs, may be involved in the intercellular movement of SCMV. Notably, ScTET78 and ScTET96 were localized in the PM and showed a punctate structure (**Figure 5**), resembling plasmodesmata. However, upon interaction with SCMV-6K2, the punctate structure disappeared (**Figure 7C**). Hence, further verification using plasmodesmata markers, such as aniline blue, is warranted. In addition, except for ScTET96, the other eight TETs exhibited both self-interaction and interaction with one another (**Figure 8**), indicating that they can form TEMs and perform various biological functions (Charrin et al., 2002; Boavida et al., 2013; Huang et al., 2022). ScTET13, ScTET23, ScTET34, ScTET55, and ScTET67 were localized in the PM, similar to some TETs in *Arabidopsis* and rice (Zhu et al., 2022). Interestingly, ScTET2 and ScTET8 form vesicular structures with diameters ranging from 0.5 to 20 μm in the cytoplasm, and both are associated with the PM (**Figure 5**). Under stressful conditions, plants produce stress granules (SGs) and P-bodies (Kearly et al., 2024). SGs are biphasic assemblies consisting of dense cores (∼0.2 μm in diameter) embedded within a less concentrated dynamic shell of variable size (Youn et al., 2019). P-bodies range from 0.8 to 1.0 μm and are located proximal to the PM (Xu and Chua, 2009), whereas MVBs range from 0.4 to 0.5 μm (Movahed et al., 2019). TET is involved in the formation of secretory vesicles and MVBs, which are employed by TuMV to move to neighboring cells (Movahed et al., 2019; Ghossoub et al., 2020; Jankovičová et al., 2020). Based on the vesicle size, we speculated that ScTET2 and ScTET8 are involved in the formation of secretory vesicles and MVBs and subsequently in cell-to-cell movement. As SG and P-bodies are membrane-less organelles, ScTET2 and ScTET8 may not be involved with them; however, further experiments are needed to verify this. AtTET8 in *Arabidopsis* serves as a marker for EVs (Zhang et al., 2020; He et al., 2021). Although ScTET2 and ScTET8 belong to Group 4 along with AtTET8, EVs range from 0.05 to 0.15 μm in diameter (Nolte-’t Hoen et al., 2016; Jeppesen et al., 2019), making them too small to be accurately distinguished by confocal microscopy due to the low resolution. Therefore, further studies using transmission electron microscopy or other techniques are necessary. *ScTET2* and *ScTET8* are highly expressed in sugarcane and show differential expression patterns upon SCMV infection. However, upon interaction with SCMV-6K2 on the PM, the vesicles induced by ScTET2 or ScTET8 disappeared. Plants can transport sRNAs through their vesicles (Li et al., 2024; Zhao et al., 2024). Therefore, we speculated that viral infection interferes with the secretory system, thereby influencing the growth and development of sugarcane plants.

Additionally, the Y2H assays showed that 17 NbTETs and 4 AtTETs interacted with TuMV-6K2 (**Supplementary Figures S10**, S11), indicating that the interaction of TET with 6K2 may represent a general mechanism employed by potyviruses to establish infection. Notably, ScTET55 and ScTET67 from sugarcane Group 3 did not interact with SCMV-6K2, indicating that TETs may be selectively utilized by different viruses.

## 5 Conclusion

In this study, 35, 113, 73, and 17 TETs were identified in *S.* sp*ontaneum* AP85-441, sugarcane cultivars XTT22 and R570, and *N. benthamiana*, respectively. These TETs clustered into nine phylogenetic groups, with Groups 7 and 8 being specific to monocotyledonous plants. The TET structure in Group 5 was significantly different from that of the other groups. Transcriptomic analysis revealed that Group 4 *TETs* were highly expressed in XTT22 and *S.* sp*ontanerum*. The nine cloned *ScTETs* from XTT22 showed different expression patterns upon SCMV infection. Subcellular localization analysis indicated that seven ScTETs were localized to the PM, with ScTET78 and ScTET96 forming punctate structures and ScTET2 and ScTET8 forming spherical structures of varying sizes. Interactions and self-interactions occurred extensively among the nine ScTETs. Seven of the nine ScTETs interacted with SCMV-6K2, and 17 N*. benthamiana* NbTETs and 4 *Arabidopsis* AtTETs interacted with the 6K2 protein of TuMV.

## Data Availability

The original contributions presented in the study are included in the article/**Supplementary Material**. Further inquiries can be directed to the corresponding author.

## Supplementary material

Supplementary Figure 1 Simulation of the structures of TETs from different species. AtTET1 (*Arabidopsis thaliana*, NP_199482.1), OsTET1 (*Oryza sativa*, LOC_Os01g74570), CD63 (Homo sapiens, AHI51903.1), as well as L6D (Homo sapiens, NP_004608.1) and ScTSPAN18 (*Saccharum* spp. Hybrid, QHD26891).

Supplementary Figure 2 Gene structure and conserved motif analysis of *TETs* genes in *Saccharum* species. **(A)** Conserved motifs of *TET* genes (labeled 1–10) are indicated by differently colored boxes. **(B)** Structure analysis of *TET* genes. Green boxes represent CDS, yellow boxes represent UTR, and black lines represent intron.

Supplementary Figure 3 Interchromosomal collinearity relationship analysis of TETs in sugarcane cultivar R570. Red lines represent the TET homologous gene pairs.

Supplementary Figure 4 Chromosomal mapping analysis of *TETs* in sugarcane cultivar *XTT22*. Blue represents chromosomes and red represents the localization of *TET* genes on chromosomes of *XTT22.*

Supplementary Figure 5 Chromosomal mapping analysis of *TETs* in *S.* sp*ontaneum*. Blue color represents chromosomes and red color represents the location of *SsTET* genes on chromosomes.

Supplementary Figure 6 Chromosomal mapping analysis of *TETs* in sugarcane cultivar *R570*. Blue color represents chromosomes and red represents the localization of *TET* genes on chromosomes of *R570.*

Supplementary Figure 7
*Cis-*acting analysis of the *TETs* in sugarcane cultivar XTT22, R570, and *S.* sp*ontanerum*. **(A)** Distribution of different *cis-*acting. **(B)** Statistic analysis of different *cis-*acting. Differently colored boxes represent different *cis-*acting.

Supplementary Figure 8 The expression patterns of *SsTETs* in different tissues or across leaf gradients of *S.* sp*ontanerum*. **(A)** The expression patterns of *SsTET* genes based on FPKM across leaf gradients in *S.* sp*ontaneum*. **(B)** The expression patterns of *SsTET* gene family based on FPKM in different tissues of different developmental stages in *S.* sp*ontaneum.*

Supplementary Figure 9 Size of vesicle like structures of localized by ScTET2-GFP in **Figure 5**. Image J was used to measure the diameter of the vesicle like structures as indicated by white arrows. Bar=20 μm.

Supplementary Figure 10 Interaction of Arabidopsis AtTETs with the 6K2 protein of turnip mosaic virus (TuMV) by Y2H assays. pPR3-AtTETs were individually pairwise co-transformed with pBT-STE-TuMV-6K2 into the yeast NMY51 cells in a 10×dilution series of 10-μL aliquots which were then plated on a non-selective medium, SD/-Leu/-Trp or quadruple dropout medium, SD/-Leu/-Trp/-His/-Ade. Yeast cells co-transformed with pTUS2-APP and pNubG-Fe65 were used as a positive control, while yeast cells co-transformed with pTUS2-APP and pPR3-N were used as negative controls.

Supplementary Figure 11 Interaction of *N. benthamiana* NbTETs with the 6K2 protein of TuMV by Y2H assays. pPR3-NbTETs were individually pairwise co-transformed with pBT-STE-TuMV-6K2 into the yeast NMY51 cells in a 10×dilution series of 10 μL aliquots which were then plated on a non-selective medium, SD/-Leu/-Trp or quadruple dropout medium, SD/-Leu/-Trp/-His/-Ade. Yeast cells co-transformed with pTUS2-APP and pNubG-Fe65 were used as a positive control, while yeast cells co-transformed with pTUS2-APP and pPR3-N were used as negative controls.
