# Supplementary figures and images for: Identification of the tetraspanin gene family in sugarcane and its response to sugarcane mosaic virus infection
Source: Front Plant Sci. 2025 Oct 27;16:1684431. doi: 10.3389/fpls.2025.1684431 (PMC12598048; doi:10.3389/fpls.2025.1684431)

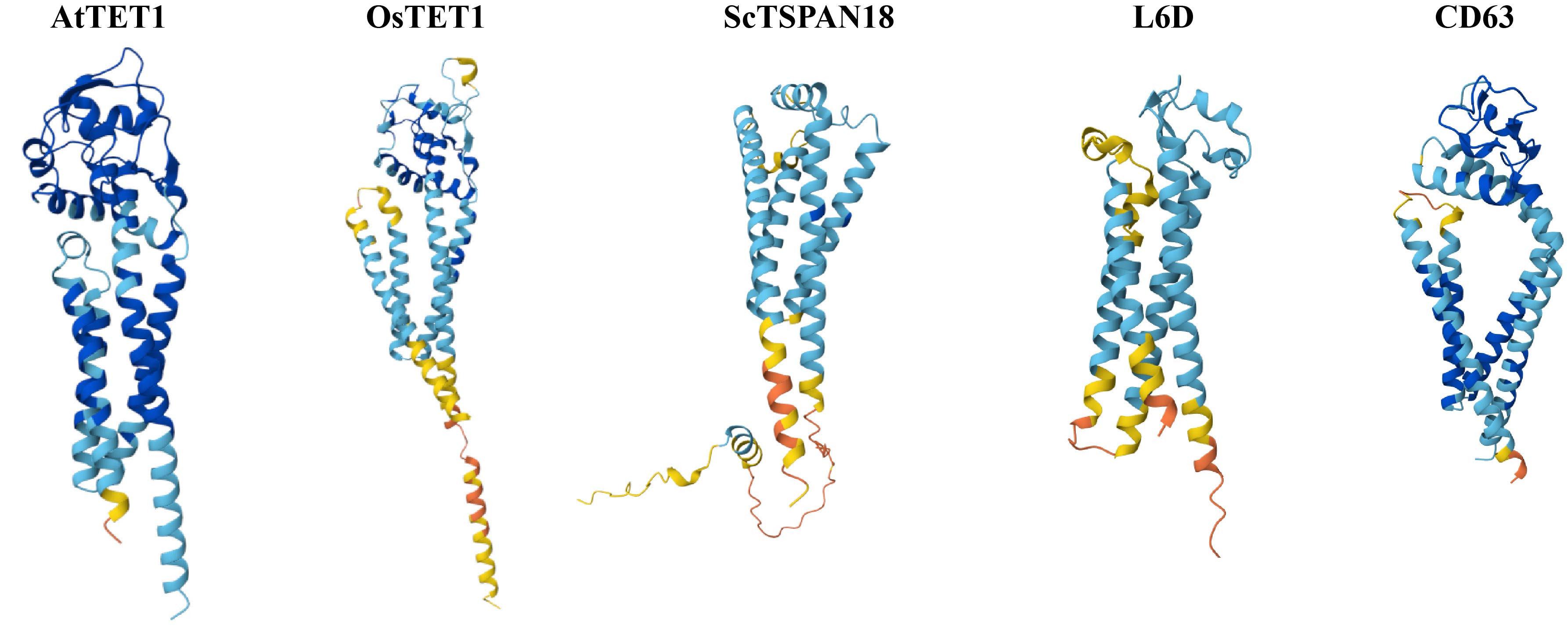

Supplement: Supplementary Figure 1 — Simulation of the structures of TETs from different species. AtTET1 (Arabidopsis thaliana, NP_199482.1), OsTET1 (Oryza sativa, LOC_Os01g74570), CD63 (Homo sapiens, AHI51903.1), as well as L6D (Homo sapiens, NP_004608.1) and ScTSPAN18 (Saccharum spp. Hybrid, QHD26891). [file Image1.jpeg]

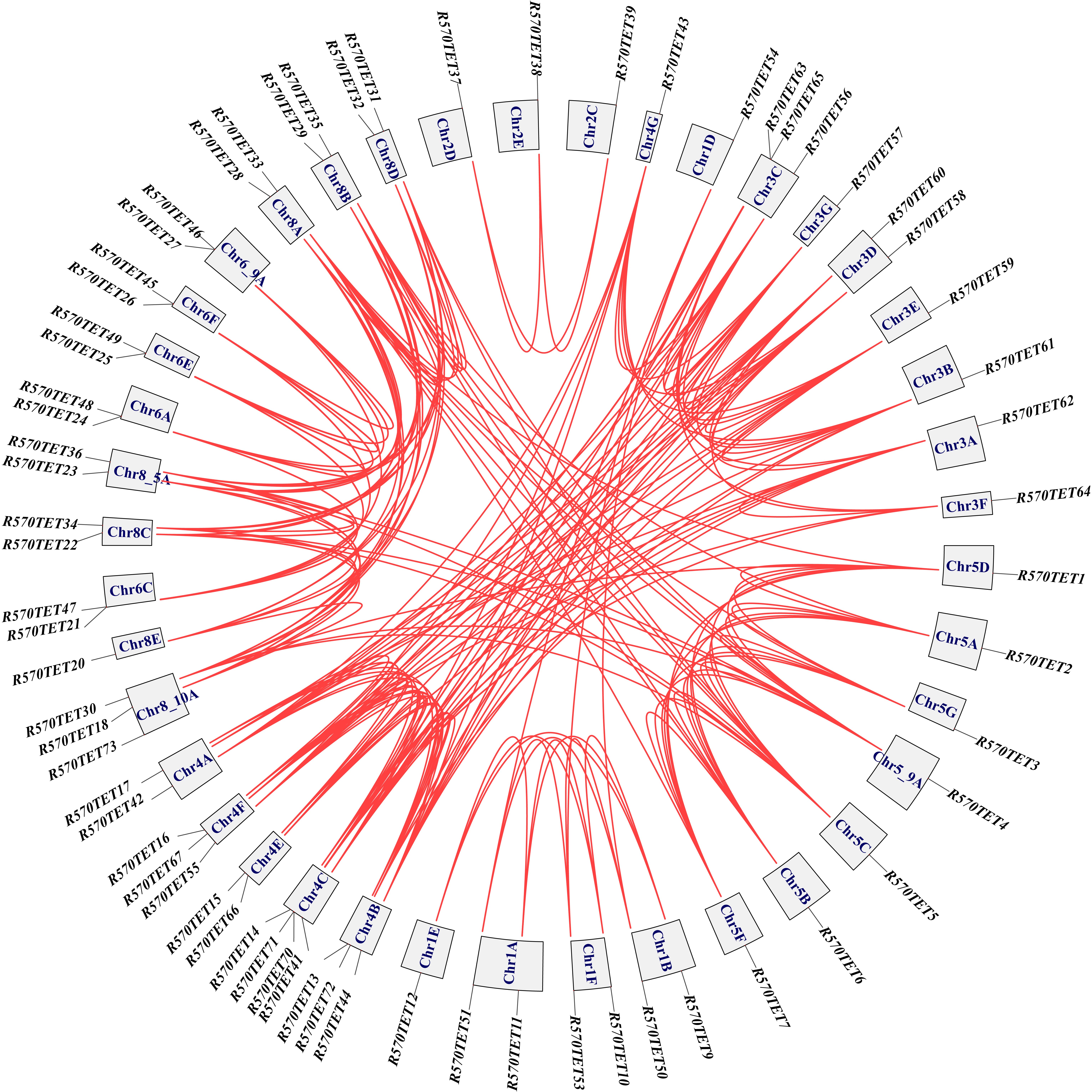

Supplement: Supplementary Figure 3 — Interchromosomal collinearity relationship analysis of TETs in sugarcane cultivar R570. Red lines represent the TET homologous gene pairs. [file Image3.jpeg]

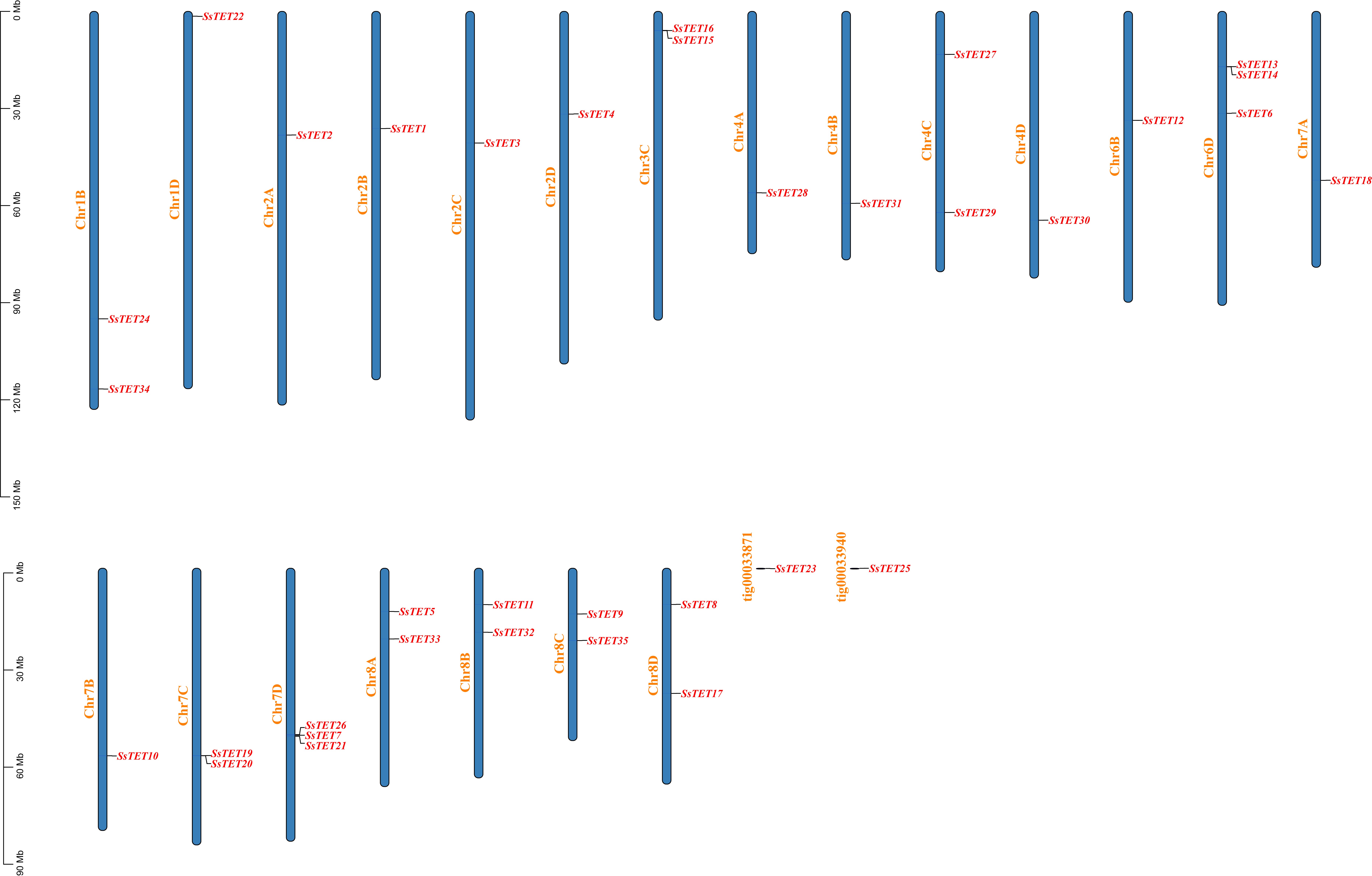

Supplement: Supplementary Figure 5 — Chromosomal mapping analysis of TETs in S. spontaneum. Blue color represents chromosomes and red color represents the location of SsTET genes on chromosomes. [file Image5.jpeg]

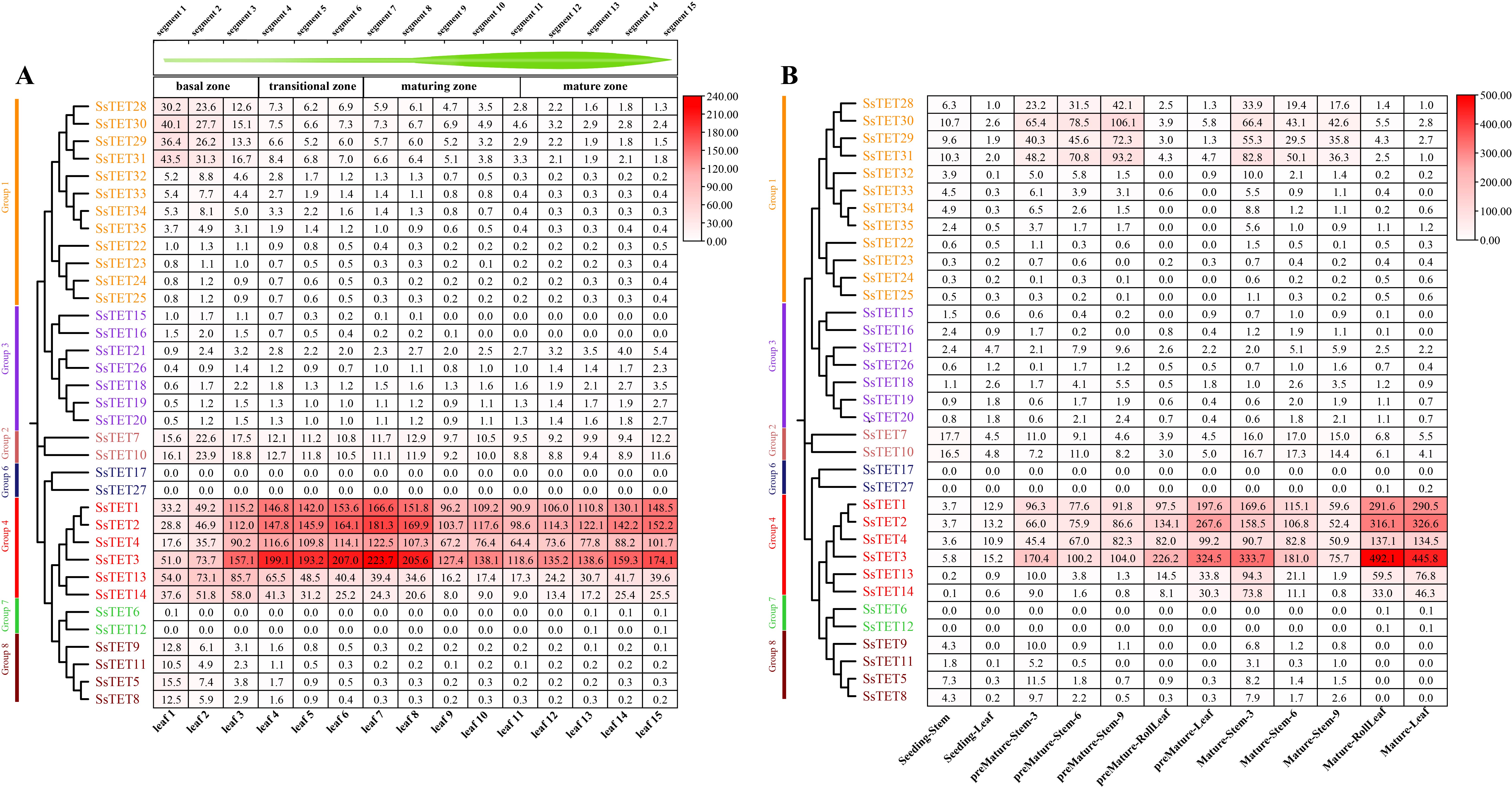

Supplement: Supplementary Figure 8 — The expression patterns of SsTETs in different tissues or across leaf gradients of S. spontanerum. (A) The expression patterns of SsTET genes based on FPKM across leaf gradients in S. spontaneum. (B) The expression patterns of SsTET gene family based on FPKM in different tissues of different developmental stages in S. spontaneum. [file Image8.jpeg]

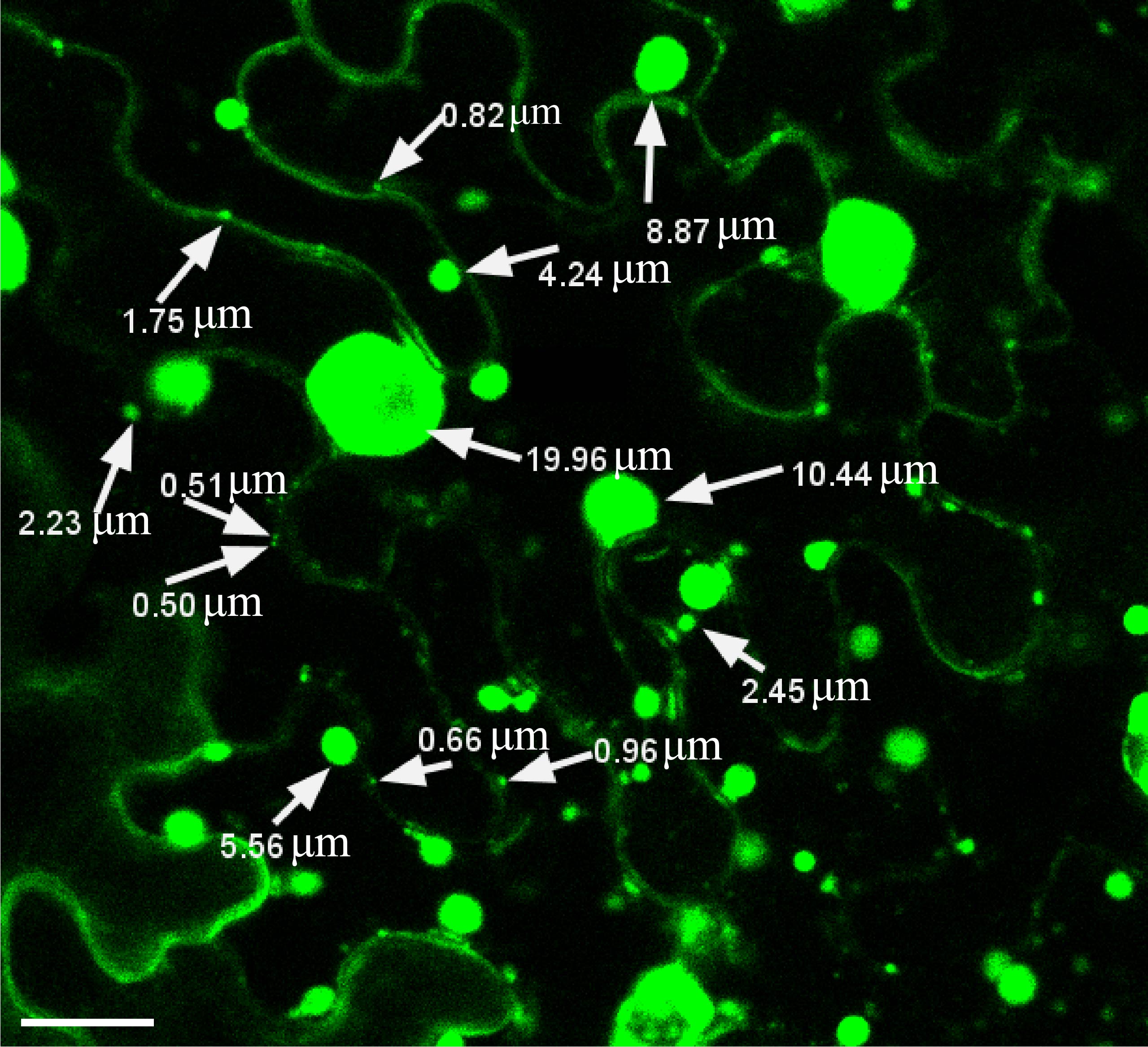

Supplement: Supplementary Figure 9 — Size of vesicle like structures of localized by ScTET2-GFP in Figure 5 . Image J was used to measure the diameter of the vesicle like structures as indicated by white arrows. Bar=20 μm. [file Image9.jpeg]

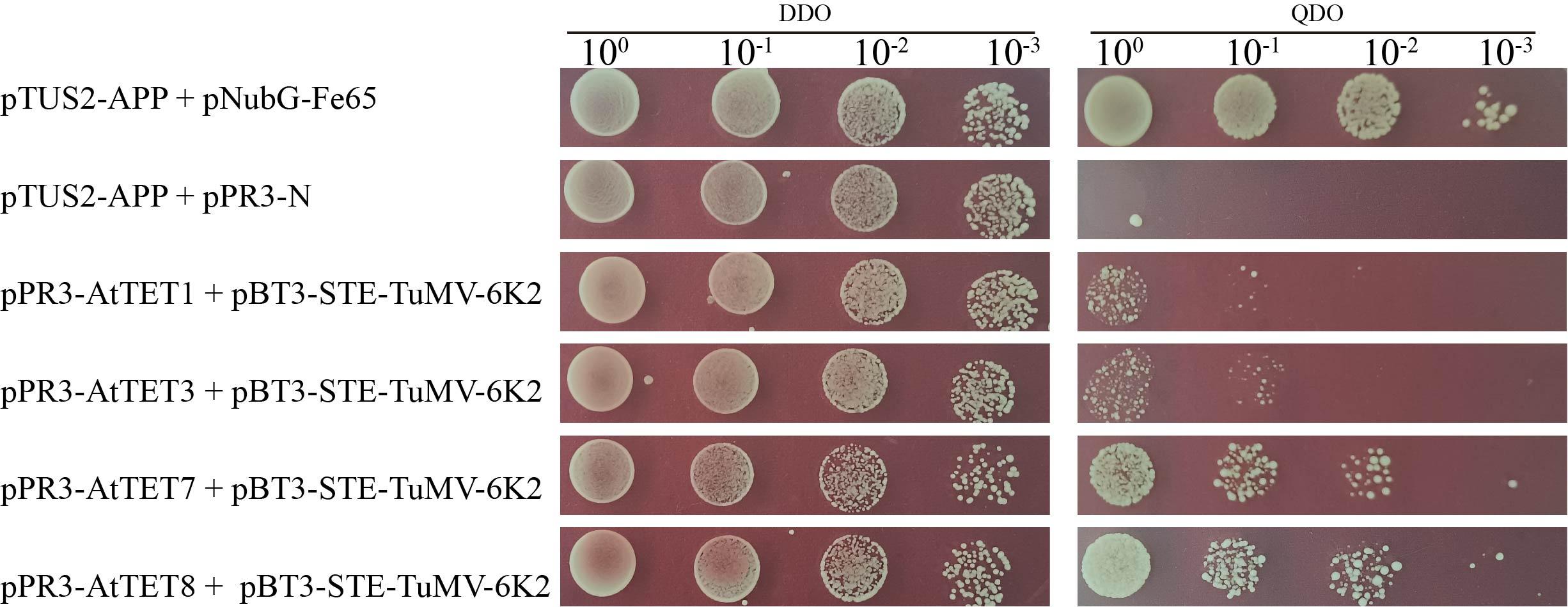

Supplement: Supplementary Figure 10 — Interaction of Arabidopsis AtTETs with the 6K2 protein of turnip mosaic virus (TuMV) by Y2H assays. pPR3-AtTETs were individually pairwise co-transformed with pBT-STE-TuMV-6K2 into the yeast NMY51 cells in a 10×dilution series of 10-μL aliquots which were then plated on a non-selective medium, SD/-Leu/-Trp or quadruple dropout medium, SD/-Leu/-Trp/-His/-Ade. Yeast cells co-transformed with pTUS2-APP and pNubG-Fe65 were used as a positive control, while yeast cells co-transformed with pTUS2-APP and pPR3-N were used as negative controls. [file Image10.jpeg]
